# Disinfection of Needleless Connector Hubs: Clinical Evidence Systematic Review

**DOI:** 10.1155/2015/796762

**Published:** 2015-05-14

**Authors:** Nancy L. Moureau, Julie Flynn

**Affiliations:** ^1^PICC Excellence, Inc., Online Education, Hartwell, GA 30643, USA; ^2^Greenville Hospital System, Greenville, SC 29605, USA; ^3^Alliance for Vascular Access Teaching and Research (AVATAR group) Griffith University, Nathan, Brisbane, QLD 4111, Australia; ^4^Royal Brisbane & Women's Hospital, Brisbane, QLD 4029, Australia

## Abstract

*Background*. Needleless connectors (NC) are used on virtually all intravascular devices, providing an easy access point for infusion connection. Colonization of NC is considered the cause of 50% of postinsertion catheter-related infections. Breaks in aseptic technique, from failure to disinfect, result in contamination and subsequent biofilm formation within NC and catheters increasing the potential for infection of central and peripheral catheters. *Methods*. This systematic review evaluated 140 studies and 34 abstracts on NC disinfection practices, the impact of hub contamination on infection, and measures of education and compliance. *Results*. The greatest risk for contamination of the catheter after insertion is the NC with 33–45% contaminated, and compliance with disinfection as low as 10%. The optimal technique or disinfection time has not been identified, although scrubbing with 70% alcohol for 5–60 seconds is recommended. Studies have reported statistically significant results in infection reduction when passive alcohol disinfection caps are used (48–86% reduction). *Clinical Implications*. It is critical for healthcare facilities and clinicians to take responsibility for compliance with basic principles of asepsis compliance, to involve frontline staff in strategies, to facilitate education that promotes understanding of the consequences of failure, and to comply with the standard of care for hub disinfection.

## 1. Background

Intravenous catheters and those related devices used to gain access to the veins for the purpose of infusing medications or solutions have evolved significantly over the past three decades. One of the more noticeable changes involves the way intravenous devices are accessed. Early concerns over needle safety for healthcare workers led to the creation of products that provide needle-free access. While these products did eliminate the risk of accidental needle injury for the clinician, some needleless products raised new issues for the patient; namely, a noted increase in the occurrence of catheter associated bloodstream infections (CABSI) and central line associated bloodstream infections (CLABSI) [1–3]. Risk factors for infection include poor adherence to aseptic technique, needleless connector (NC) design variations, and inconsistent health care staff education and training [1–3]. NC are used on virtually all intravascular devices in the USA; they provide an easy access point for syringe or tubing attachment and have now become the central access point for all connections. Yet, despite providing some level of safety, concerns over infection related to NC contamination exist. Surface design, gaps around valve closure surface, segmented fluid pathway with dead space, differing internal mechanisms, clear or obscured visibility, variable blood reflux, clamping sequences, and different flushing instructions, depending on the type of NC, all play a part in the level of risk associated with the device. Before the advent of NC, clinicians had an intuitive understanding that prior to penetrating the septum with the needle the septum required disinfection. Current surface disinfection of NC is not necessarily intuitive. Initially, needleless split septum access points used a blunt “needle-looking” type cannula. As a result, the disinfection process remained intuitive. Split septum access devices continue to be recommended as a lower risk option for needleless connection; however, they have lost popularity because they require multiple parts and pieces for access and allow direct needle access through the septum/diaphragm leading many facilities to switch to luer access devices. With the changes to the access point using direct luer connection through the NC, the intuitive sense to disinfect the surface prior to access is lost; many clinicians fail to realize the consequences of this breech in aseptic technique [4–6]. Colonization of catheter hubs and NC, with subsequent bacterial ingress into the catheter lumen, is considered the cause of 50% of postinsertion catheter-related infections [3–7]. Disinfection of the exposed surface of the NC is necessary to avoid contamination and subsequent intraluminal biofilm formation and protect patients from infection.

Vast improvements have been made in the reduction of CLABSIs attributed to insertion procedures. The results of the groundbreaking Keystone initiative demonstrated the effect of five measures, known as the Central Line Bundle, on the improvement of outcomes during insertion of central venous catheters [64, 105]. Consistent application of the bundle, with compliance verified during the insertion procedure (checklist), has reduced insertion related CLABSI by more than 44% in the USA [52]. However, despite the successes of the insertion bundle, full compliance more than seven years later is still lacking, with reported compliance rates at one institution ranging from 0.0% at the beginning of the intervention to 37.1% (139/375), according to the Jeong study, with similar results in other institutions [65, 66, 106, 107]. Even in institution where full compliance of the bundle exists, CLABSIs are still occurring [108]. Disinfection of the NC access site was not included in the insertion related central line bundle. The goal of any effective infection prevention program is zero CLABSIs. To reach the goal of zero, consideration must be given for the pathogenesis of catheter related infections and an investigation into current human factors of catheter management preventing achievement of this goal.

While many experts agree that application of the insertion bundle is one of the best ways to prevent insertion-related infection, the bundle does not address NC, aseptic access, or any postinsertion catheter usage issues. A Pennsylvania study reported that 71.7% (468/653) of central line infections occurred five days or more after insertion and may have been directly related to use and care of intravascular devices [60, 108–110]. Contamination of the catheter directly through the catheter hub has been confirmed through published studies [12, 13, 111–113]. These studies found that bacteria identified on external hub surfaces were also present in biofilm sampled from random locations within the needless connector. Research performed at one institution revealed that patient skin flora was not the source of catheter related bloodstream infections in any of their cases; all infections in this study originated from the catheter hub [6, 113]. Infections later in the life of the catheter develop from improper catheter manipulation, failure to perform hand hygiene, inadequate time to clean NC, inadequate training, and poor access and exit site management [2, 67, 110, 112]. Disinfection of a catheter hub prior to flushing or prior to the administration of medications is required for all aseptic access, yet in the Karchmer study, 31% of clinicians did not even attempt to disinfect, even when under active observation [1, 64, 88, 114]. In a study by Lee the disinfection compliance by clinicians prior to NC access was measured at only 10% [115]. This common break in aseptic technique sets the stage for biofilm formation within NC and catheters and increases the potential for delayed infection of both central and peripheral catheters [14, 60, 68, 112, 116]. The results of the Pennsylvania Patient Safety Advisory Report and independent biofilm sampling of NC suggest that more attention is needed for aseptic access and maintenance practices [109].

## 2. What Is Disinfection?

According to the Epic3 Evidence-Based Guidelines for Preventing Healthcare Infections, disinfection is defined as the use of chemical or physical methods to reduce the number of pathogenic microorganisms on surfaces to a level at which they are not able to cause harm, but which does not usually destroy spores [53]. These guidelines further state that disinfection methods used in combination with cleaning blood or other debris off the surface as disinfectants have limited ability to penetrate organic material [8]. The Association for Professionals in Infection Control (APIC) defines disinfection as a process to eliminate microorganisms accomplished with the use of liquid chemicals or pasteurizing; process works best by having proper contact time and dilution of disinfection agent [117]. Recommendations from the Centers for Disease Control [8], the Agency for Healthcare Research and Quality [9], the Society for Healthcare Epidemiology of America [10], and the Infusion Nurses Society [11, 118] state that NC should be consistently and thoroughly disinfected using mechanical friction with 70% alcohol, alcoholic chlorhexidine, or povidone iodine prior to each access of an intravascular device and listed in evidence as a Category 1A.

### 2.1. Goal

The goal of this review is to assess current literature related to disinfection of NC to establish recommendations that promote aseptic access, reducing infection risk for the patient.

### 2.2. Search Methodology

The purpose of this systematic review was to evaluate the supporting evidence for disinfection practices of NC, catheter hub, stopcock, and side ports that reduce the transfer of microorganisms through intravascular device access. This report is based on an electronic systematic literature search and review of published materials from Pubmed, Medline, Scopus, Ovid, jStor, CINAHL, Cochrane, Athens, and ScienceDirect by cross-referencing these key terms for years 1977–December 2014. High level evidence from RCTs that tested “cause and effect” relationships between different disinfection approaches for NC and patient infection was initially sought. Since no RCTs were found, lower level evidence including clinical and in vitro (laboratory) studies was reviewed, as long as these included reporting of quantitative data. Broad MeSH search term “disinfection” and “needleless connector” combinations were used with additional keywords listed below:

- disinfection, antiseptic, alcohol, chlorhexidine, and anti-infective agents,
- intravenous, intravascular, and vascular access,
- hub, catheter hub, scrub the hub,
- intravenous connector, NC, luer activated device, and mechanical valve,
- aseptic practices, contamination, and compliance,
- education, staff education, and medical education,
- infection, infection prevention, catheter related infections, CLABSI, bloodstream infections, bacteremia, sepsis, and cross-infection,
- catheter maintenance and line care,
- insertion and bundle,
- intravenous technology,
- catheter cap, access port, disinfecting cap, antimicrobial cap, hub protection cap, and port protector,
- Infection prevention guidelines and recommendations.

Additional studies were cross-referenced through manual search. Conference posters and abstracts were included in the review. Manufacturers websites of disinfection ports and two manufacturers (Excelsior Medical, Neptune, NJ; Ivera Medical Corporation, San Diego, CA) were contacted directly requesting all published materials and posters on disinfection products. There were no identified formal published systematic reviews of the effectiveness of NC disinfection practices, indicating a knowledge gap in this area. Search results were evaluated by title, abstract, and content. Selected papers were subjected to full-text assessment. Initial selection process and critique was performed by one researcher (NM), with evidence rating performed by two researchers independently (NM and JF), with any disagreement in quality rating resolved by discussion.

### 2.3. Inclusion/Exclusion Criteria

Criteria checklist for inclusion was any NC disinfection publications and abstracts that fit subcategories for disinfection, hub contamination/infection prevention, education/compliance, surveys, and guidelines/recommendations for disinfection. Inclusion criteria consisted of publications meeting search terms and topic requirements under sub groupings:

1. disinfection agents used on intravascular device surfaces including studies and reviews of NC and infection prevention,
2. sources of contamination through intravascular devices,
3. education and compliance for infection prevention,
4. guidelines and recommendations for infection prevention with disinfection.

Exclusion criteria were

- nonresearch papers,
- studies of adult, pediatric, or neonatal increasingly important role patients not inclusive of intravascular device disinfection practices,
- primary populations outside acute care,
- publications not translated into English,
- studies prior to 1984.

## 3. Results

**Figure 1:**
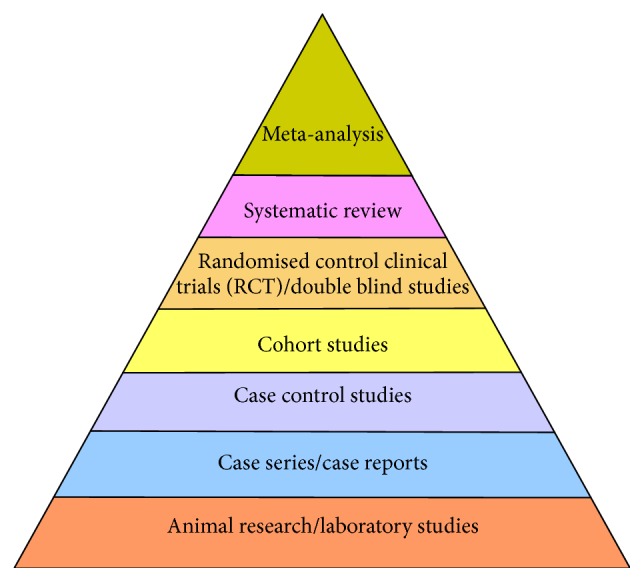


The systematic review of these topics yielded a total of 433 papers and abstracts. After initial review 259 articles did not meet eligibility requirements and were removed. Included studies consisted of 140 publications dealing with disinfection/catheter hub/NC contamination with 34 abstracts/posters. Of the studies 67 were graded according to the strength of the study. The study results and ratings of the literature are included in Tables 1–5 and Figure 1, with recommendations are represented in Table 6.

### 3.1. Why Disinfect?

A catheter is inserted into a vein or artery to provide a pathway for the administration of medications or solutions necessary to improve a patient's health or condition. Because catheters provide an open conduit into the vasculature, a NC is attached, via luer threaded connection, to the integrated hub end of the catheter establishing a closed system. Studies reflecting benefits of closed systems with NC have trended toward demonstration of protection for catheter and hub colonization [4, 119]. In a prospective controlled study by Rosenthal and Maki and multicenter prospective cohort by Rangel-Frausto et al., open systems compared to closed systems resulted in major reductions in catheter related infections [120]. NC used as a closed system must be weighed with consideration for potential negative factors of design features, poor aseptic practices, and lack of disinfection that all contribute to risk of infection [2, 121, 122].

Any puncture through the protective skin barrier creates a portal for bacteria to enter the body. Recognized routes of catheter contamination are classified as either extraluminal or intraluminal and include (a) migration of microorganisms from the skin at the insertion site (considered the source in short term infections), (b) catheter hub contamination, (c) hematogenous seeding from another infection source in the body, and (d) direct contamination from an infusate [8, 108, 123]. After insertion of a catheter, introduction of microorganisms occurs primarily from two routes: the skin/insertion track or through the lumen of the catheter [15, 124–127]. The greatest risk for contamination of the catheter after insertion is the access hub with 33–45% (402/900) contaminated in normal patient use [6, 15, 128–132]. In early studies by Sitges-Serra colonization of the catheter hub was considered the primary pathogenesis of catheter associated infection [15, 113]. Linares and colleagues reported 14 episodes of sepsis (70% of total catheter related septic events) resulted from hub-related contamination [127, 133]. Moro reported hub colonization in only 3.5% (21/607), but found that this group was responsible for severe systemic infections more frequently [128]. Studies indicate that, during periods of nonuse, colony forming units (CFU) are present on access hubs in numbers ranging from 15 to 1000 CFU, representing quantities sufficient to cause contamination, biofilm formation, and potentially bacteremia if not sufficiently disinfected prior to access [3, 5, 12, 99, 110, 111, 134–143]. As demonstrated by multiple studies, infections are drastically lower or eliminated by disinfecting or covering the access hub with an antimicrobial cap [14, 16–20, 113, 127, 144–146]. Hub contamination plays an increasingly important role with infection risk the longer the catheter is in place [15]. Intraluminal contamination and subsequent colonization become more prominent with longer dwell times [110, 147]. Perez and associates found 59% (42/75) of one group of NC colonized with biofilm and Salzman found that 71% (20/28) of catheter related infections originated in the catheter hub presumably from contamination [15, 21, 22, 148]. Clearly hub contamination is a causative element in catheter related infections and one that demonstrates the necessity for effective hub disinfection prior to access [110, 113, 127, 133, 144, 149].

### 3.2. What to Disinfect?

Disinfection points to gain access to intravenous or intravascular devices may include tubing side ports, direct catheter connections, stopcocks with needle free caps, NC of various types (split septum, mechanical valves, positive pressure valves, zero, or neutral connectors), traditional silicone septum, or other forms of access integrated with the catheter or tubing. Any intravascular access point with a surface open to the environment requires disinfection prior to use, as it acts as the immediate portal of entry for intraluminal contaminants [23, 99, 113, 127, 133, 144, 150–152]. Needle free devices constitute more than 80% of access devices, are recommended by Centers for Disease Control for all tubing/catheter access, and are now more common than traditional covered septal access ports which allowed needles to pass easily through the silicone or rubber covered access [8, 153]. Primary areas of focus for disinfection of access sites are the point where the sterile syringe or tubing contacts the site, as in the top septum surface, and the threads or side surfaces [7, 143, 154]. Manufacturers are required to include instructions for device use and disinfection recommendations with each product to guide in the correct and safe usage of the NC.

Effective disinfection of a NC is influenced by several factors including: ability to clean the NC surface, the amount and position of grooves or gaps present, and the roughness or smoothness of the septum [1, 3, 7, 69, 150, 154–156]. All NC consist of a septum, a fluid pathway and a mechanism for activation; the design, space, volume, and human factors all affect how easy a product is to use and disinfect, and may also act as contributors to the potential risk of catheter associated bloodstream infection [3, 69, 150, 155, 157, 158]. NC have gaps of differing widths between the septal seal and the housing which may allow ingress of microorganisms [7, 23, 99, 143]. Adequate cleaning or need for additional cleaning of the septal access site may be based on the specific design features of the individual NC [2, 4, 5, 7, 70, 111, 129, 153, 159–161]. New products or technology should be transitioned into a healthcare facility only after a complete evaluation of both the research and the performance of the product to determine the impact of the change on patient outcomes [71, 97, 98, 149, 160, 162–166].

### 3.3. Disinfection Practices

Recommendations from both the Centers for Disease Control and the Infusion Nurses Society state clinician should minimize contamination risk by disinfection the access ports of the add-on device using friction with an appropriate disinfectant (70% alcohol, chlorhexidine, povidone iodine, and iodophors) prior to any access [8, 11, 24, 167, 168]. The 70% isopropyl alcohol wipe is most commonly used to disinfect the access surface of NC and has been proven effective or ineffective at disinfection times from 5 seconds to 60 seconds [14, 19, 24–27, 131, 151, 169–177]. The major biocide effect of alcohol occurs while wet and immediately after drying, allowing for dehydration of bacterial cells, whereas alcoholic chlorhexidine is most effective during the drying process, where it enters the cell to cause destruction providing ongoing antimicrobial effect [150, 172]. The disinfecting action of chlorhexidine in combination with alcohol, allowing for both immediate and sustained action; has proven to be more effective than either agent alone [21, 131, 168, 172, 176, 178–180]. Faster drying time with alcohol makes it superior to other disinfection agents and provides an advantage to chlorhexidine when used in combination. Effectiveness of 70% alcohol disinfection is variable based on application techniques and characteristics of NC surface and design, leading some researchers to conclude that complete disinfection of microorganisms on some NC surfaces may not be achievable [12]. In the Menyhay prospective in vitro study, 20 (67%) of 30 NC disinfected with 70% alcohol resulted in transmission of contaminants (442–25,000 CFU) yet 60 tested with barrier caps (containing 2% chlorhexidine and 70% alcohol) showed only one (1.6%) with transmission of contaminants [19]. In both the Kaler laboratory and the Ruschman randomized experimental design studies, using a 15 second and 60 second scrub respectively, disinfection with 70% alcohol eliminated all microorganisms [173, 181]. Kaler performed laboratory testing on contaminated NC with a small sample using 70% alcohol and alcoholic chlorhexidine and found both to be effective for hub disinfection [181]. Two additional studies gave conflicting results. Rupp demonstrated 5 second alcohol disinfection was effective; this was in direct contrast with the Smith study where contact time of 10/12/15 seconds was deemed adequate, but 5 and 8 seconds were not as effective to prevent bacterial transfer [170, 171]. Simmons and colleagues found 3/10/15 seconds significantly decreased the bacterial load in an in vitro laboratory study, with some level of bacteria remaining during all duration levels tested; disinfection failed to completely eliminate contaminants [13]. More studies are needed to provide efficacy for optimal time necessary to eliminate surface contaminants.

Research of Macias and associates with 2% chlorhexidine in 70% isopropyl alcohol on skin proved an added substantive effect, even against freshly introduced organisms, for up to 24 hours, establishing this agent as a superior disinfecting agent when longer action is needed, in comparison with single agents of 70% isopropyl alcohol, 10% povidone iodine and 10% sodium hypochlorite [172]. Alcoholic chlorhexidine performed consistently well or better than other disinfection agents in multiple studies [21, 24, 131, 168, 172, 179, 182]. In the research by Hong et al., a 5 second scrub with alcoholic chlorhexidine fully disinfected NC surfaces treated with Pseudomonas Aeruginosa [179]. In the most recent Epic3 United Kingdom report of evidence-based guidelines, recommendations by expert consensus include a 15 second cleansing with alcoholic chlorhexidine prior to and after each access [53]. In actual practice disinfection prior to access is expected, while cleansing after each access is rarely done.

### 3.4. Passive Disinfection

Disinfection research includes various forms of passive antimicrobial hub protection with 70% alcohol caps (SwabCap, Excelsior Medical, Neptune, NJ; Curos Port Protector, Ivera Medical, San Diego, CA;, EffectIV-Cap, Hospira, Lake Forest, IL; DualCap, Catheter Connections, Salt Lake City, UT), iodinated alcohol hub, povidone iodine gauze and specialty covers, and combination chlorhexidine/alcohol caps [12, 14, 18, 19, 23, 25–43, 146, 178, 183–192]. In a randomized prospective trial by Pittiruti, 46 catheters received a 70% alcohol port protector with no detected CLABSIs over 707 catheter days, colonization in two catheters and no contaminated blood cultures [18]. Of note the Pittiruti study resulted in reductions of CLABSIs in the port protector/disinfection cap group and the control group, with improvements attributed to both the disinfection caps and educational efforts. These disinfection caps applied and left in place provide active mechanical friction along with longer contact time creating a physical and chemical barrier between the lumen and the environment [26]. As a progressive CLABSI intervention Posa at St. Joseph Mercy Health System implemented an insertion bundle, chlorhexidine bathing, a maintenance bundle, chlorhexidine dressing for central catheters, and educational programs, however it was not until implementing the 70% alcohol disinfection cap that their rates of CLABSI fell to zero and remained from 2011 to the end of 2012 The disinfection cap placed on all access ports eliminates human factor issues requiring clinicians to remember to carry the necessary disinfection supplies to the bedside or even to remember to perform the act of disinfection before each access [187]. An in vivo hospital study by DeVries gave nurses a choice to use either this single use cleansing cap or a disinfection cap to leave on the NC access site, clinicians preferred the longer lasting disinfection cap [14]. In another retrospective study, Schears noted a predisinfection cap CLABSI rate of 1.682/1000 catheter days and a CLABSI rate of 0.6461/1000 catheter days after implementing disinfection caps, representing a statistically significant 61% reduction in CLABSI [32]. In Wright et al.'s study at NorthShore University HealthSystem, a four University Hospital system, the intervention with 70% alcohol disinfection caps reported CLABSI rates declining from 1.42/1000 catheter days (16/11,540) to 0.69 (13/18,972) with a 95% confidence interval, based on 799 enrolled patients, representing a statistically significant decrease [26]. Another alcoholic hub protector study by Sweet et al. included 472 patients and 3005 catheter days and showed a decrease in overall CLABSIs from 2.3 to 0.3/1000 catheter days and a PICC CLABSI reduction from 2.3 to 0, a statistically significant change, with an 85.2% compliance rate [31]. Stango and associates reported a 50% reduction in CLABSIs and a savings of $464,440 per year after alcoholic cap implementation [184]. Numerous studies have demonstrated consistent clinical effectiveness of 70% alcohol caps alone in studies and abstracts graded C or D [18, 26, 27, 31, 33, 41, 43, 178, 189, 191–194]. Alcoholic chlorhexidine caps are also effective in preventing contamination and completely disinfecting NC access surfaces (combination of alcoholic chlorhexidine in cap form is not commercially available in the USA) [2, 12, 19, 178, 179].

Another engineered solution for hub cleaning involves a 70% alcohol foam cap (Site Scrub, Bard Access, Salt Lake City, UT) designed for use as an access site cleansing cap for single use, then discarded. The Holroyd in vitro study at University of Florida compared the single use of this cleansing cap with 70% alcohol to traditional 70% alcohol wipes [151]. When the cleansing cap was used on stopcocks Holroyd found contamination and increased CFU. This study found 70% alcohol wipes and this alcohol cleansing cap were both effective on the surface of NC and catheter hubs [151]. Other groups also used this single use cleansing cap in combination with other 70% alcohol disinfecting caps designed to be left in place until the next access [14, 177].

### 3.5. Clinical Implications

Since the advent of NC as access hubs for the administration of medications or fluids, there has been a need to verify compliance with disinfection practices prior to access. During the period of needle usage for catheter access, nurses and doctors intuitively knew the necessity of disinfecting the access septum prior to inserting a needle. With NC, these questions arise: is disinfection always performed prior to access? Is disinfection performed in an effective manner? Do clinicians fully understand the consequences of not performing disinfection? Disinfection practices with alcohol or alcoholic chlorhexidine that include adequate contact disinfection time are effective if performed at all. According to a recent publication by Ryder, an issue was raised regarding whether failure to disinfect is considered a medical error and if so, is this omission considered negligence? [100] Catheter associated infections are a significant safety issue, and contamination caused by lack of aseptic technique is preventable. Once contamination occurs, bacteria attach to the inner lumen of the catheter, begin to grow and form biofilm, making successful eradication extremely difficult [6, 28–30, 113, 127, 133, 140–142, 144, 195]. Joint Commission now requires hospitals in the USA to protect patients by having a standard and measurable protocol for hub/access site disinfection [61, 62, 196]. Measurement of compliance with hub disinfection is challenging, requiring direct observation of the action unless disinfection caps/ports are used on all NC hubs as a form of verification. Passive disinfection through hub protectors/disinfection caps have differing designs and colors, leading to easy recognition and validation of compliance with usage. Reimbursement structures in the USA that now promote pay for performance and penalize poor outcomes will assist in driving these passive safety strategies that aid in monitoring and improving compliance with disinfection.

### 3.6. Issues of Compliance and Monitoring

While policies for disinfection of access devices are a first step, methods to validate actual practice and patient safety must be integrated into hospital culture. The central line bundle checklist is used as evidence to demonstrate compliance with safety practices during insertion, but the aspect of day to day management is not addressed in the bundle. Care and management of catheters takes up more than 99% of the dwell time of a catheter compared to the one hour or less for catheter insertion. Compliance with aseptic practice is important for both insertion and daily usage. Consistent hand hygiene and gloving performed prior to any procedure or even touching of a catheter helps reduce bacterial transfer. Application of alcoholic chlorhexidine to disinfect skin for central line insertions and now, more and more with peripheral catheter insertions, is helping to reduce bacterial ingress to the bloodstream. Maximum sterile barriers also reduce contamination during the insertion process so that overall, CLABSI rates occurring in the first few days of insertion continue to fall.

Even with the success of the Central Line Bundle on CLABSI reductions, a majority of hospitals remain well above zero for infections. Access and maintenance activities with the catheters may be to blame. When CLABSIs occur well after the 96 hour mark, contamination of the catheter through the NC is likely the culprit. From the evidence presented, NC and catheter hubs are a primary source of bacterial contamination, and subsequent transmission of contamination into the catheter lumen [6, 30, 113, 127, 133, 142, 144]. Just one omission of scrubbing the hub prior to access permits bacterial entry, attachment and biofilm formation that allow the bacteria to strengthen prior to release into the bloodstream. Preventing this form of contamination requires teaching and constant reinforcement of the required practice of regular and consistent disinfection prior to every access. Verification of compliance with hub disinfection by clinicians requires direct observation of the action unless disinfection hub protectors are used, providing a form of passive immediate visual verification. More and more studies are demonstrating lack of compliance with hub disinfection despite educational initiatives and better disinfection agents. Disinfection methods that incorporate prolonged duration of contact with an antiseptic agent to significantly decrease the level of surface bacteria present may provide a solution to the problem of hub contamination and variation in NC designs.

Various studies provide statements regarding conformity or lack of conformity concerning disinfection practices, attributing noncompliance to a lack of universal protocols, excessive workloads (e.g., when clinicians become busy, they are less likely to comply), or just forgetting to bring alcohol wipes to the bedside [54, 64, 72, 89–93, 188]. The Smith study on behavioral intention indicated a negative correlation between performing optimal disinfection with increasing age of clinicians and more years of experience [91]. Clearly there are human factors working against disinfection of hubs prior to access requiring engineered solutions such as passive disinfecting cap strips hanging on intravenous pump poles, supply dispensers of alcohol wipes at the bedside, or on the intravenous pump to ensure greater, even 100% compliance with disinfection each and every time [91, 170]. Monitoring and validation of hub disinfection compliance is necessary to determine if other measures are needed such as disinfection caps/port protectors. In an evaluation of 5877 physicians, nurses and technicians, Jardim et al. documented compliance with hub disinfection 38.7% of the time, leaving more than 61% of accesses without disinfection, leading to possible contamination and biofilm growth [92]. Platace et al. evaluated clinician hands during invasive procedures demonstrating 100% of the 48 nurses sampled exceeded acceptable levels of microorganisms, with the potential to contaminate and cause bloodstream infection [90]. Studies show there is a need for clear recommendations and practices that prevent transmission of contaminants through NC [68, 90]. Targeting of education for providers responsible for CVAD insertion and care for identifying appropriate indications, performing insertion with the central line bundle, performing surveillance of CLABSI and scrubbing the hub with an appropriate antiseptic are Category IA recommendations by AHRQ as critical components of a comprehensive CLABSI prevention program [9].

### 3.7. Risk of Bias and Limitations

This systematic review highlights the lack of available high quality research in this area that tests the cause and effect relationship between NC disinfection practices and patient infection outcomes. It also asks the question “What are we basing our clinical practice guidelines on for disinfection of NC?” Absence of high quality RCT evidence required authors to include any clinical observational and cohort studies and laboratory studies. Overall, the evidence base for the effectiveness of various disinfection strategies is low level, resulting in recommendations compiled from the available publications. The strength of this review is that it includes all relevant, currently available pieces of evidence; however there remains a high level of uncertainty in the estimates of effectiveness of various decontamination techniques, and these are highly likely to change with the publication of new studies in the literature. Studies to date have a risk of unintentional bias due to the lack of randomization and control groups/strategies, in addition to small sample sizes and retrospective study designs. Randomized controlled studies are needed to rigorously evaluate the efficacy of disinfection practices and antiseptic hub protectors in preventing patient infection.

### 3.8. Research Priorities

Adequately large randomized controlled trials are urgently needed to establish high quality evidence of the efficacy of various disinfection practices to prevent infection. Randomized controlled trials are needed to identify if risk reduction differs with the type of antiseptic, for example, 70% alcohol versus 70% alcohol and chlorhexidine, or with differing concentrations of chlorhexidine in their efficacy for disinfection NC. Research may also validate the substantive effect of alcoholic chlorhexidine on NC and its continued antimicrobial activity on these surfaces, potentially establishing a reduced cleaning frequency or duration for NC. Study considerations for passive disinfection coupled with prefilled flush syringes could demonstrate drastic reduction of hub contamination and intraluminal biofilm colonization, but ultimately patient infection outcomes are needed. Research that replicates solid studies provides a stronger foundation for evidence-based practice and should be encouraged. Translational research is growing, providing clinical implications that directly apply to bedside practices [14, 57–59, 67, 70, 73–75]. NC disinfection is an excellent subject for efficacy studies utilizing comparative research for various disinfection approaches identifying relative reductions in patient infection risk. Claire Rickard, Ph.D., Professor of Nursing with Griffith University, states it well “We are belatedly realising that to eliminate these complications (infections) we must conduct research, implement evidence-based interventions and reduce the clinical practice variations that lead to their occurrence” [197]. Research and study are necessary as an integral part of professional practice, providing a means to direct clinical activity and to share with rising young clinicians long after we are gone.

## 4. Conclusion

Aseptic technique is the foundation for safe delivery of intravenous medications and solutions. More and more studies reveal lack of compliance with disinfection of access ports prior to and after access, despite educational initiatives, and better disinfection agents [1, 26, 27, 38, 54, 57, 64, 67, 69, 72, 74, 78, 83, 88–93, 188, 198–205]. Rather than creating devices such as the ultraviolet C port to eradicate contamination within the hub, the goal should be to eliminate surface pathogens before entering the NC or catheter. Passive disinfection caps reduce guess work, provide clinicians with a point of use solution, and reduce contamination. It is critical for healthcare facilities and clinicians to take responsibility for compliance with aseptic technique for NC disinfection, to monitor compliance regularly, to involve frontline staff in solutions, and to facilitate education that promotes understanding of the consequences of failure to comply with the standard of care for access site disinfection.

## Figures and Tables

**Table 1:** Disinfecting agents and devices literature.

	Grade^*^
(1) J. Bak et al., *Photochem Photobio*, vol. 87, pp. 1123–1128, 2011.	D
(2) J. Bak and T. Begovic, *J Hosp Infect*, vol. 84, pp. 173–177, 2013.	D
(3) J. D. Brown, H. A. Moss, and T. S. Elliott, *J Hosp Infection, *vol. 36, pp. 181–189, 1997.	D
(4) A. L. Buchman, J. Spapperi, and P. Leopold, *J Vasc Access, *vol. 10, no. 1, pp. 11–21, 2009.	D
(5) A. L. Casey et al., *J Hosp Inf, *vol. 54, no. 4, pp. 288–293, 2003.	B
(6) C. Chernecky, L. Casella, E. Jarvis et al., *J Research Nsg*, vol. 15, no. 5, pp. 405–415, 2010.	D
(7) M. DeVries, P. S. Mancos, and M. J. Valentine, *J Assoc Vasc Access*, vol. 19, no. 2, pp. 87–93, 2014.	C
(8) K. Field, C. McFarlane et al., *Infect Control Hosp Epidemiol, vol. 28, no. 5, pp. 610–613, 2007. *	C
(9) P. Gould and A. Oudakker, “Getting to ZERO central line associated bloodstream infections,” *Poster AVA*, National Harbour, M.d., USA, September 2010.	C
(10) K. Guerin, J. Wagner, K. Rains, and M. Bessesen, *Am J Infect Control*, vol. 38, no. 6, pp. 430–433, 2010.	C
(11) J. L. Holroyd, D. A. Paulus et al., *Anesth Analg, *vol. 118, no. 2, pp. 333–343, 2010.	D
(12) H. Hong, D. F. Morrow, T. J. Sandora, and G. P. Priebe, *Am J Infect Control*, vol. 41, no. 8, pp. e77–e79, 2013.	D
(13) W. Kaler and R. Chinn, *JAVA*, vol. 12, no. 3, pp. 140–147, 2007.	D
(14) J. P. Kennedy, R. A. Lasher, D. Solomon, and R. W. Hitchcock, *J Medical Devices*, vol. 4, no. 2, Article ID 027509, 2010.	D
(15) C. Leon, F. Alvarez-Lerma, S. Ruiz-Santana et al., *Crit Care Med*, vol. 31, no. 5, pp. 1318–1324, 2003.	D
(16) M. Leone and L. Dillon, *J Infusion Nsg*, vol. 31, no. 2, pp. 84–91, 2008.	C
(17) M. Leone and M. Pratt, *Infusion*, pp. 10–13, Nov/Dec 2011.	D
(18) J. L. Lockman, E. S. Heitmiller, J. A. Ascenzi, and I. Berkowitz, *Anesth*, vol. 114, p. 958, 2011.	D
(19) R. W. Loftus et al., *Anesthesia*, vol. 115, no. 5, pp. 1109–1118, 2012.	D
(20) J. Luna, G. Masdeu et al., *Eur J Clin Micro Infect Dis*, vol. 19, pp. 655–662, 2000.	C-D
(21) J. Macias et al., *Am J Infect Control*, vol. 31, pp. 634–637, 2013.	D
(22) D. G. Maki, *Clinical Infectious Diseases*, vol. 50, no. 12, pp. 1580–1587, 2010.	D
(23) M. Mazher et al., *Letters in Applied Microbiology*, vol. 57, no. 4, pp. 282–287, 2013.	D
(24) S. Z. Menyhay and D. G. Maki, *Infect Control Hosp Epidemiol*, vol. 27, no. 1, pp. 23–27, 2006.	D
(25) S. Z. Menyhay and D. G. Maki, *Am J Infect Control*, vol. 36, no. 10, pp. S174.e171–S174.e175, 2008.	D
(26) K. C. Merrill et al., *Am J Infect Control*, vol. 42, no. 12, pp. 1274–1277, 2014.	B-C
(27) M. K. Muffly et al., *Am J Infect Control*, vol. 38, no. 9, pp. 734–739, 2010.	D
(28) J. Oto, H. Imanaka, M. Konno, E. Nakataki, and M. Nishimura, *Am J Infect Control*, vol. 39, no. 4, pp. 309–313, 2011.	B-C
(29) E. Perez et al., *Journal of Clinical Microbiology*, vol. 52, no. 3, pp. 823–831, 2014.	B-C
(30) C. Ramirez, A. Lee, and K. Welch, *JAVA*, vol. 17, no. 4, pp. 210–213, 2014.	B-C
(31) M. E. Rupp, S. Yu, T. Huerta et al., *Infect Control Hosp Epidemiol*, vol. 33, no. 7, pp. 661–665, 2012.	C
(32) K. L. Ruschman and J. S. Fulton, *J Intraven Nurs*, vol. 16, no. 5, pp. 304–308, 1993.	D
(33) C. Salgado et al., *Infect Control Hosp Epidemiol*, vol. 28, no. 6, pp. 684–688, 2007.	C
(34) M. Salzman, H. Isenberg, and L. Rubin, *J Clin Microbiol*, vol. 31, no. 3, pp. 475–479, 1993.	D
(35) S. Sannoh et al., *Am J Infect Control*, vol. 38, no. 6, pp. 424–429, 2010.	C
(36) M. Segura, F. Alvarez-Lerma, J. M. Tellado et al., *Ann Surg*, vol. 223, no. 4, pp. 363–369, 1996.	D
(37) S. Simmons, C. Bryson, and S. Porter, *Critical Care Nursing Quarterly*, vol. 34, no. 1, pp. 31–35, 2011.	D
(38) J. Smith, G. Irwin, M. Viney et al., *J Assoc Vasc Access*, vol. 17, no. 3, 2012.	D
(39) J. S. Soothill, K. Bravery, A. Ho, S. Macqueen, J. Collins, and P. Lock, *Am J Infect Control*, vol. 37, no. 8, pp. 626–630, 2009.	C
(40) C. Stango, D. Runyon, J. Stern, I. Macri, and M. Vacca, *JIN*, vol. 37, no. 6, pp. 1–4, 2014.	C
(41) M. A. Sweet, A. Cumpston, F. Briggs, M. Craig, and M. Hamadani, *Am J Infect Control*, vol. 40, no. 10, pp. 931–934, 2012.	C-B
(42) M. Wright, J. Tropp, D. Schora et al., *Am J Infect Control*, vol. 41, no. 1, pp. 33–38, 2012.	C-B
(43) J. C. Yebenes, M. Delgado, G. Sauca et al., *Crit Care Med*, vol. 36, no. 9, pp. 2558–2561, 2008.	D

^*^Grade of recommendation was modified from the NHMRC definitions (NHMRC, 2009) [102]. To achieve a grade of A the research is required to be a high quality randomized control trial (RCT) or a systematic review of high quality RCTs. Laboratory (in vitro) research was classified as level V evidence (DeVries and Berlet, 2010 [103]; The University of Newcastle Australia, 2014 [104]).

A: body of evidence can be trusted to guide practice, systematic review or RCT.

B: body of evidence can be trusted to guide practice in most situations, RCT or high quality observational study.

C: body of evidence provides some support for recommendation but care should be taken in its application, observational studies.

D: Level V evidence or evidence that is weak and recommendation must be applied with caution, expert opinion, animal or laboratory studies.

**Table 2:** Needleless connector literature.

	Grade^*^
(1) E. Bouza et al., *J Hosp Infect, *vol.54, no. 4, pp. 279–287, 2003.	B
(2) B. Caillouet, *J Assoc Vasc Access, *vol.17, no. 2, pp. 86–89, 2012.	C
(3) D. Cain and G. Jones, “Comparison of catheter occlusions between a mechanical valve injection cap and positive displacement injection cap,” *Poster NHIA*, Dallas, Tex, USA, April 12–15, 2010.	C
(4) A. L. Casey, S. Burnell et al., *J Hosp Infect,* vol. 65, no. 3, pp. 212–218, 2007.	B-C
(5) A. L. Casey, T. Worthington, P. A. Lambert, D. Quinn, M. H. Faroqui, and T. S. Elliott, *J Hosp Infect, *vol. 54, no. 4, pp. 288–293, 2003.	B-C
(6) C. Chernecky and J. Waller, *J Adv Nsg,* vol. 67, no. 7, pp. 1601–1613, 2011.	D
(7) C. C. Chernecky, D. Macklin, W. R. Jarvis, and T. V. Joshua, *AJIC*, vol. 42, no. 2, pp. 200–202, 2014.	B-C
(8) S. Cicalini, F. Palmieri, and N. Petrosillo, *Critcal Care*, vol. 8, pp. 157–162, 2004.	B-C
(9) J. M. Costello, D. F. Morrow et al., *Pediatrics*, vol. 121, pp. 915–923, 2008.	C
(10) ECRI Institute, “Evaluation: needleless connectors,” *Health Devices, *vol. 37, no. 9, pp. 261–281, 2008.	D
(11) C. E. Edmiston, V. Markina, *AJIC*, vol. 38, pp. 421–423, 2010.	D
(12) F. Esteve, M. Pujol, E. Limon et al., *Journal of Hospital Infection, *vol. 67, no. 1, pp. 30–34, 2007.	B-C
(13) Hadaway L., *J Assoc Vasc Access*, vol. 16, no. 1, pp. 20–33, 2011.	D
(14) M. Ishizuka, H. Nagata, K. Takagi, and K. Kubota, *Int Surg*, vol. 98, pp. 88–93, 2013.	C
(15) W. Jarvis, C. Murphy, K. Hall et al., *Clin Infect Dis*, vol. 49, no. 12, pp. 1821–1827, 2009.	C
(16) N. Khalidi, D. S. Sovacevich, L. F. Papke-O'Donnell, and I. Btaiche, *J Assoc Vasc Access*, vol 14, no. 2, pp. 84–91, 2009.	C
(17) B. S. Niël-Weise, T. J. Daha, P. J. van den Broek, *J Hosp Infect*, vol. 62, no. 4, pp. 406–13, 2006.	A
(18) C. Salgado, L. Chinnes, T. Paczesny, and J. Cantey, *Infect Control Hosp Epidemiol, *vol. 28, no. 6, pp. 684–688, 2007.	C
(19) S. Schilling, D. Doellman, N. Hutchinson, and B. R. Jacobs, *J Paren Ent Nut, *vol. 30, no. 2, pp. 85–90, 2006.	B-C
(20) R. J. Sherertz, T. B. Karchmer, E. Palavecino, and W. Bischoff, *European J Clin Micro Infect Dis*, vol. 30, no. 12, pp. 1571–1577, 2011.	C
(21) L. Steininger, “In search of zero: eight years of interventions lead to reduced central line associated bloodstream infection rates,” *Poster 5th Decennial International Conference on Healthcare-Associated Infections*, Organized by SHEA, CDC, APIC, and IDSA, Atlanta, Ga, USA, March 2010.	C
(22) Y. P. Tabak, W. R. Jarvis, X. Sun, C. T. Crosby, and R. S. Johannes, *Am J Infect Control*, vol. 42, no. 12, pp. 1278–1284, 2014.	A
(23) J. C. Yébenes, R. Martínez, M. Serra-Prat et al., *Am J Infect Control*, vol. 31, no. 8, pp. 462–464, 2003.	D
(24) J. C. Yébenes, L. Vidaur, M. Serra-Prat, J. M. Sirvent, J. Batlle, M. Motje, A. Bonet, and M. Palomar, *Am J Infect Control*, vol. 32, no. 5, pp. 291–295, 2004.	B

^*^Grade of recommendation was modified from the NHMRC definitions (NHMRC, 2009) [102]. To achieve a grade of A the research is required to be a high quality randomized control trial (RCT) or a systematic review of high quality RCTs. Laboratory (in vitro) research was classified as level V evidence (DeVries and Berlet 2010 [103]; The University of Newcastle Australia, 2014 [104]).

A: body of evidence can be trusted to guide practice, systematic review or RCT.

B: body of evidence can be trusted to guide practice in most situations, RCT or high quality observational study.

C: body of evidence provides some support for recommendation but care should be taken in its application, observational studies.

D: Level V evidence or evidence that is weak and recommendation must be applied with caution, expert opinion, animal, or laboratory studies.

See Figure 1.

**Table 3:** Poster and abstract presentations on disinfecting caps/port protectors.

	Year
(1) F. Alasmari, N. Kittur, A. Russo et al., “Impact of alcohol impregnated protectors on incidence of catheter-associated blood stream infections,” *IDSA Poster*, 2012.	2012
(2) T. Antony and M. Levin, “MacNeal Hospital: engineered device dramatically improves efficacy leading to fewer CLABSIs,” *AVA Annual Scientific Meeting*, National Harbor, M.d., USA, 2010.	2010
(3) B. Bor, C. Johnson, and C. Noble, “It takes a village to prevent central venous catheter infections and promote safety of patients,” *AVA Annual Scientific Meeting*, San Antonio, Tex, USA, 2012.	2012
(4) C. Chernecky, “Biofilm formation in connectors characterized by using electron microscopy,” Paper presented at: Association for Vascular Access Scientific Meeting, National Harbor, M.d., USA, September 2014.	2014
(5) M. Cole and K. Kennedy, “Grady health system: decreasing central line associated blood stream infections (CLABSI) in adult ICUs through teamwork and ownership,” *GHA Patient Safety Summit*, Atlanta, Ga, USA, 2013.	2013
(6) H. Contreras, “Use of disinfection cap/flush syringe combination to address bloodstream infection and related issues,” *AVA Annual Scientific Meeting*, San Antonio, Tex, USA, 2012.	2012
(7) B. Danielson, S. Williamson, G. Kaur et al., “Decreasing the incidence of central line-associated blood stream infections using alcohol-impregnated port protectors (AIPPS) in a neonatal intensive care unit,” *40th Annual Conference Abstracts, APIC 2013*, Ft. Lauderdale, Fla, USA, 2013.	2013
(8) M. Davis, “Forcing the function: implementation and evaluation of an IV port protector to decrease CLABSI,” *National Teaching Institute & Critical Care Exposition*, 2013.	2013
(9) R. Dawson and N. Moureau, “Implementing new joint commission requirements using revised protocol to disinfect intravenous access ports/needleless connectors,” *12th Annual NPSF Patient Safety Congress*, Orlando, Fla, USA, 2010.	2010
(10) M. DeVries, P. Mancos, and M. Valentine, “Improving catheter cleaning and maintenance in central and peripheral lines,” *APIC Annual Conference*, Ft. Lauderdale, Fla, USA, 2013.	2013
(11) A. Dobin, “Broward Health Coral Springs Medical Center: bloodstream infections eliminated by use of a plastic cap for disinfecting needleless connectors,” 2010.	2010
(12) L. Fink, “What to do when ‘scrubbing the hub' does not work,” *APIC Poster Presentation*, Presentation no. 2–246, 2013.	2013
(13) T. Karchmer, E. Cook, E. Palavecino, C. Ohl, and R. Sherertz, “Needleless valve ports may be associated with a high rate of catheter-related bloodstream infection,” *Abstract 15th Annual Scientific Meeting of the Society for Healthcare Epidemiology of America*, Los Angeles, Calif, USA, 2005.	2005
(14) G. Kaye, “Weiss Memorial Hospital: new disinfection cap achieves joint commission compliance for valve disinfection not achievable with alcohol prep pads,” *AVA Annual Scientific Meeting*, National Harbor, M.d., USA, 2010.	2010
(15) J. Kelleher, R. Almeida, H. Cooper, and S. Stauffer, “Hospital PSHMCaCs. Achieving zero CoN CLBSI in the NICU,” *APIC Annual Conference*, Ft. Lauderdale, Fla, USA, 2013.	2013
(16) J. Lee, “Disinfection cap makes critical difference in central line bundle for reducing CLABSIs,” *APIC Annual Conference*, Ft. Lauderdale, Fla, USA, 2011.	2011
(17) B. Lopansri, I. Nicolescu, J. Parada, A. Tomich, J. Belmares, and P. Schereckenberger, “Microbial colonization of needleless intravenous connectors and the male luer end of IV administration sets: does the partner matter?” *SHEA Annual Scientific Meeting*, Dallas, Tex, USA, 2011.	2011
(18) D. Maslak, D. Rossettini, L. Trento, and M. Leone, “Catheter maintenance in the home parenteral nutrition patient = reduced CRBSIs,” *Infusion Nursing Society Annual Convention*, Poster Presentation, Phoenix, Ariz, USA, 2014.	2014
(19) S. McCalla, J. Greco, M. Warren, P. Byrne, and J. Bogetti, “Integrated delivery system of disinfection cap and flush syringe, plus staff education, reduce bloodstream infections and treatment costs,” *AVA Annual Scientific Meeting*, San Antonio, Tex, USA, 2012.	2012
(20) M. Moore, K. Gripp, H. Cooper, and R. Almeida, “Providence Sacred Heart Medical Center: impact of port protectors on incidence of central line infections,” *APIC Annual Conference*, Ft. Lauderdale, Fla, USA, 2013.	2013
(21) N. Moureau and R. Dawson, “Passive disinfection product effectiveness study,” AVA Annual Scientific Meeting, Poster Presentation, National Harbor, M.d., USA, September 24–26, 2010.	2010
(22) M. Pavia, “Testing elimination of an infection prevention device from catheter bundle and potential effect on overall catheter bloodstream infection rate,” *APIC Annual Conference*, vol. 41, no. 6, p. S36, Ft. Lauderdale, Fla, USA, 2013.	2013
(23) M. Pittiruti, “Port protectors and educational intervention: the key to zero central line-associated bloodstream infection: a randomized controlled trial,” *Association for Vascular Access Scientific Meeting*, Poster Abstract Presentation, National Harbor, M.d., USA, September 7–10, 2014.	2014
(24) A. Pong, C. Salgado, M. Speziale, P. Grimm, and C. Abe, “Rady Children's Hospital San Diego, reduction of central line associated bloodstream infection (CLABSI) in a neonatal intensive care unit with use of access site disinfection caps,” *IDSA Annual Meeting*, Boston, Mass, USA, 2011.	2011
(25) P. Posa, “Improving IV connector disinfection by using human factors engineering to identify effective, nurse-friendly solutions,” *APIC Annual Conference*, Ft. Lauderdale, Fla, USA, 2014.	2014
(26) M. Pratt and M. Leone, “Coram specialty infusion services. An evaluation of the effectiveness of intravenous disinfection caps in the prevention of CVAD infections in parenteral nutrition,” *A.S.P.E.N. Clinical Nutrition Week Conference*, Vancouver, Canada, 2011.	2011
(27) M. Ryder, “Access site and hub disinfection: in vitro testing of a novel device,” *SHEA Annual Scientific Meeting*, Poster Presentation, abstract 168, 2009.	2009
(28) G. Schears, “Cap the connector: save the patient,” *AVA Annual Scientific Meeting*, 2011.	2011
(29) R. Seiler and S. Meyer, “Improving infection control compliance using combined cap/flush syringe technology to reduce central line associated bloodstream infections,” *AVA Annual Scientific Meeting*, Nashville, Tenn, USA, 2013.	2013
(30) J. Shiber, G. Jolicoeur, and T. Crouchet, “Ochsner Medical Center: reducing central line-associated bloodstream infections through the addition of disinfecting port protector caps to the central line bundle,” *Annual Research Day*, New Orleans, La, USA, May 2014.	2014
(31) E. Simpser, H. Painter, and M. Pavia, “St Marys Healthcare System for Children: reducing central line-associated bloodstream infections through additions to bundle,” *NACHRI Annual Leadership Conference*, Bellevue, Wash, USA, 2011.	2011
(32) M. Small, “St Marks Hospital: The effect of 70% isopropyl alcohol port protection on central venous catheter related infection in patients on home parenteral nutrition,” *World Congress Vascular Access, WOCOVA 2014*, Berlin, Germany, 2014.	2014
(33) P. Stokes, “St Francis Hospital: device for disinfecting needleless connectors solves problem of inconsistent swabbing technique,” *AVA Annual Scientific Meeting*, San Jose, Calif, USA, 2011.	2011
(34) S. Sumner, K. C. Merrill, L. Linford, and C. Taylor, “Decreasing CLABSI rates and cost following implementation of a disinfectant cap in a tertiary care hospital,” *40th Annual Conference Abstracts, APIC 2013,* Ft. Lauderdale, Fla, USA, 2013.	2013

**Table 4:** Why disinfect? Sources of contamination literature.

	Year
(1) B. Brismar, L. Jordahl et al., *Clinical Nutrition*, vol. 6, no. 1, pp. 31–33, 1984.	1984
(2) I. F. Btaiche, D. S. Kovacevich et al., *Am J Infect Control*, vol. 39, no. 4, pp. 277–283, 2011.	2011
(3) A. R. Burrell, M. L. McLaws et al., *Med J Aust*, vol. 194, no. 11, pp. 583–587, 2011.	2011
(4) C. Chernecky, “Biofilm formation in connectors characterized by using electron microscopy,” *Abstract Assoc Vasc Access Scientific Conference*, National Harbor, M.d., USA, September 2014.	2014
(5) D. Cozanitis and P. Makela, *Acta anaesthesiologica Belgica*, vol. 59, no. 2, pp. 59–63, 2008.	2008
(6) J. Davis, “Pennsylvania patient safety authority 2011,” *Patient Safety Advisory*, vol. 8, no. 3, pp. 100–104, 2011.	2011
(7) R. Donlan, *Curr Top Microbiol Immunol*, vol. 322, pp. 133–161, 2008.	2008
(8) R. M. Donlan and J. W. Costerton, *Clinical Microbiology Reviews*, vol. 15, no. 2, pp. 167–193, 2002.	2002
(9) R. Donlan, *Emerging Infection Diseases*, vol. 7, no. 2, pp. 277–281, 2001.	2001
(10) L. Hadaway, *Nursing Management*, vol. 39, no. 10, p. 17, 2008.	2008
(11) L. Hadaway, *J Infus Nurs*, vol. 35, no. 4, pp. 230–240, 2012.	2012
(12) L. Hadaway, *Journal of Infusion Nursing*, vol. 26, no. 1, pp. 44–48, 2003.	2003
(13) J. Liñares, A. Sitges-Serra, J. Garau, J. Perez, and R. Martin, *Journal of Clinical Microbiology*, vol. 21, no. 3, pp. 357–360, 1985.	1985
(14) B. L. Lobo, G. Vaidean et al., *J Hosp Med*, vol. 4, no. 7, pp. 417–422, 2009.	2009
(15) J.-C. Lucet, J. Hayon et al., *Infection Control and Hospital Epidemiology*, vol. 21, no. 1, pp. 40–42, 2000.	2000
(16) M. A. Luebke, M. J. Arduino, D. L. Duda et al., *American Journal of Infection Control*, vol. 26, no. 4, pp. 437–441, 1998.	1998
(17) L. M. Mahieu, A. O. De Muynck et al., *J Hosp Infect*, vol. 48, pp. 108–116, 2001.	2001
(18) D. Macklin, J Assoc Vasc Access, vol. 15, no. 3, pp. 126–150, 2010.	2010
(19) D. Maki, C. E. Weise, and H. W. Sarafin, *New England Journal Medicine,* vol. 296, pp. 1305–1309, 1977.	1977
(20) D. Maki, *Anesth Analg*, vol. 56, no. 1, pp. 141–153, 1977.	1977
(21) L. A. Mermel, *Clinical Infectious Diseases,* vol. 52, no. 2, pp. 211-212, 2011.	2011
(22) M. L. Moro, E. F. Vigano, and A. Cozzi Lepri, *Infect Control Hosp Epidemiol,* vol. 15, no. 4, part 1, pp. 253–264, 1994.	1994
(23) I. Raad, W. Costerton et al., *Journal of Infectious Diseases, *vol. 168, no. 2, pp. 400–407, 1993.	1993
(24) I. Raad, M. Luna, S. Khalil, J. Costerton, C. Lam, and G. Bodey, *JAMA,* vol. 271, no. 13, pp. 1014–1016, 1994.	1994
(25) P. Ramritu, K. Halton, D. Cook, M. Whitby, and N. Graves, *Journal of Advanced Nursing*, vol. 62, no. 1, pp. 3–21, 2008.	2008
(26) C. M. Rickard, J. Webster, and E. G. Playford, *Med J Aust,* vol. 198, no. 10, pp. 519-520, 2013.	2013
(27) M. Ryder, G. Hamilton, M. Hamilton, and G. James, “Bacterial transfer through needlefree connectors: comparison of nine different devices,” *Society for Healthcare Epidemiology of America Annual Scientific Meeting*, Baltimore, M.d., USA, 2007.	2007
(28) M. Ryder, *Topics in Advanced Practice Nursing eJournal*, vol. 2005, 2005.	2005
(29) M. Ryder, *Advocate, *2006.	2006
(30) M. B. Salzman, H. D. Isenberg et al., *J Infect Dis,* vol. 167, no. 2, pp. 487–490, 1993.	1993
(31) M. B. Salzman and L. G. Rubin, *Nutrition, *vol. 13, supplement 4, pp. 15s–17s, 1997.	1997
(32) N. Safdar and D. Maki, *Intensive Care Med,* vol. 30, no. 1, pp. 62–67, 2004.	2004
(33) A. Sitges-Serra et al., *Nutrition,* vol 13, supplement 4, pp. 30s–35s, 1997.	1997
(34) A. Sitges-Serra, J. Linares, and J. Garau, *Surgery,* vol. 97, no. 3, pp. 355–357, 1985.	1985
(35) A. Sitges-Serra, J. Linares, J. L. Perez, E. Jaurrieta, and L. Lorente, *J Parenter Enteral Nutr,* vol. 9, no. 3, pp. 322–325, 1985.	1985
(36) A. Sitges-Serra, P. Puig, J. Linares et al., *J Parenter Enteral Nutr,* vol. 8, no. 6, pp. 668–672, 1984.	1984
(37) B. W. Trautner and R. O. Darouiche, *Archives of Internal Medicine, *vol. 164, no. 8, pp. 842–850, 2004.	2004
(38) J. M. Walz, K. Faris, S. O. Heard, *Critical Care Medicine,* vol. 36, no. 9, pp. 2689-2690, 2008.	2008

**Table 5:** Education and compliance literature.

	Year
(1) N. Arora et al., *Am J Med Qual*, vol. 29, no. 4, pp. 329–334, 2013.	2013
(2) K. Y. Blot et al., *Crit Care Med, *vol. 42, no. 5, p. e382, 2014.	2014
(3) K. Blot et al., *Clin Infect Dis*, vol. 59, no. 1, pp. 96–105, 2014.	2014
(4) C. L. Boyer, J Intraven Nurs, vol. 21, supplement 5, pp. S161–S165, 1998.	1998
(5) C. Chernecky et al., *Clin J Oncol Nurs*, vol. 13, no. 6, pp. 630–633, 2009.	2009
(6) M. G. Cherry et al., *Med Teach*, vol. 32, no. 3, pp. 198–218, 2010.	2010
(7) M. G. Cherry et al., *Med Teach*, vol. 34, no. 6, pp. e406–e420, 2012.	2012
(8) S. Cookson et al., *Infect Control Hosp Epidemiol*, vol. 19, no. 1, pp. 23–27, 1998.	1998
(9) K. Cooper et al., *J Hosp Infect, *vol. 86, no. 1, pp. 47–52, 2014.	2014
(10) C. Coopersmith, J. E. Zack, M. R. Ward et al., vol. 139, no. 2, pp. 131–136, 2004.	2004
(11) M. G. Fakih et al., *Infect Control Hosp Epidemiol, *vol. 33, no. 5, pp. 449–455, 2012.	2012
(12) B. M. Farr, *Infect Control Hosp Epidemiol, *vol. 21, no. 6, pp. 411–416, 2000.	2000
(13) G. K. Frampton, P. Harris, K. Cooper et al., *Health Technol Assess, *vol. 18, no. 15, pp. 1–365, 2014.	2014
(14) K. Herzer, L. Niessen et al., *BMJ Open*, vol. 2014, article 4, 2014.	2014
(15) Y. J. Hsu, K. Weeks et al., *Am J Infect Control, *vol. 42, supplement 10, pp. S191–196, 2014.	2014
(16) J. M. Jardim, R. A. Lacerda et al., *Revista da Escola de Enfermagem da USP,* vol. 47, no. 1, pp. 38–45, 2013.	2013
(17) I. S. Jeong, S. M. Park, J. M. Lee, J. Y. Song, and S. J. Lee, *Am J Infect Control*, vol. 41, no. 8, pp. 710–716, 2013.	2013
(18) R. D. Lobo, A. S. Levin, L. M. Gomes et al., *Am J Infect Control*, vol. 33, no. 2, pp. 83–87, 2005.	2005
(19) D. Macklin, C. Chernecky, K. Nugent, J. Waller, *Journal of Vascular Access Devices, *vol. 8, no. 2, pp. 8–13, 2003.	2003
(20) A. S. McAlearney, J. L. Hefner, *Am J Infect Control, *vol. 42, supplement 10, pp. S216–222, 2014.	2014
(21) M. McGuckin, R. Waterman, L. Porten et al., *Am J Infect Control, *vol. 27, no. 4, pp. 309–314, 1999.	1999
(22) A. Mian, C. Russell, M. Honeycutt, and C. Oldridge, *J Ark Med Soc, *vol. 109, no. 7, pp. 128–131, 2012.	2012
(23) N. Moureau, “Winning the war on CLABSIs: the role of education and new technology” *ICT, *2009.	2009
(24) D. Platace, I. Klava, L. Antonevica et al., *Acta Chirurgica Latviensis*, vol. 9, no. 1, pp. 50–55, 2010.	2010
(25) P. Pronovost, D. Needham, S. Berenholtz et al., *The New England Journal of Medicine*, vol. 355, no. 26, pp. 2725–2732, 2006.	2006
(26) P. Rangachari, *Qual Manag Health Care, *vol. 22, no. 1, pp. 16–24, 2013.	2013
(27) G. D. Sacks, B. S. Diggs et al., *Am J Surg*, vol. 207, no. 6, pp. 817–823, 2014.	2014
(28) J. Segreti, S. Garcia-Houchins, L. Gorski et al., *JIN, *vol. 34, no. 2, pp. 126–133, 2011.	2011
(29) J. S. Smith, K. M. Kirksey, H. Becker, A. Brown, *J Infus Nurs*, vol. 34, no. 3, pp. 193–200, 2011.	2011
(30) D. Warren, J. Zack, and J. Mayfield, *CHEST, *vol. 126, no. 5, pp. 1612–1618, 2004.	2004
(31) D. K. Warren et al., *Infect Control Hosp Epidemiol, *vol. 27, no. 7, pp. 662–669, 2006.	2006
(32) D. K. Warren, D. S. Yokoe, M. W. Climo et al., *Infect Control Hosp Epidemiol, *vol. 27, no. 1, pp. 8–13, 2006.	2006
(33) D. K. Warren, J. E. Zack, M. J. Cox, M. M. Cohen, and V. J. Fraser, *Critical Care Medicine, *vol. 31, no. 7, pp. 1959–1963, 2003.	2003
(34) E. Young, M. Commiskey, S. Wilson, *Am J Infect Control, *vol. 34, no. 8, pp. 503–506, 2006.	2006
(35) W. Zingg, A. Imhof, M. Maggiorini et al., *Critical Care Medicine, *vol. 37, no. 7, pp. 2167–2173, 2009.	2009

**Table 6:** 

	Recommendations for disinfecting practices	Levels of evidence^*^
1	Use disinfection on surfaces of needleless connectors, stopcocks and other intravascular access ports immediately prior to any connection, infusion or aspiration with appropriate antiseptic agent (e.g., alcoholic chlorhexidine, povidone iodine, an iodophor, or 70% alcohol). Access catheter connections with sterile devices only [8–11].	B

2	Antimicrobial caps/port protectors may be effectively used for passive continuous hub disinfection on needleless connections in accordance with manufacturer instructions, in conjunction with frictional antiseptic wiping between applications and access [2, 6, 10, 12–51].	B-C

3	Ensure compliance with hand hygiene, gloving and aseptic practices prior to any contact with intravenous devices and add-on equipment [6, 8, 10, 30, 52–59].	B

4	Establish and educate all clinical staff on a standard protocol to disinfect catheter hubs, needleless connectors and ports prior to and after each access [11, 20, 60–63].	B-C

5	Provide consistent and varied staff education on consequences of poor technique along with clinical reminders of best practice [10, 13, 51, 54, 60, 64–87].	C

6	Establish regular surveillance of compliance for disinfection of intravascular devices prior to access with reporting of results to each care unit [1, 67, 72, 78, 88–96].	C

7	Establish a formal process to evaluate new technology and needleless connector designs [7, 71, 97, 98].	A

8	Implement a multimodal quality improvement infection prevention program that applies guidelines and recommendations to all intravascular practices [68, 78, 85, 99–101].	B

^*^Grade of recommendation was modified from the NHMRC definitions (NHMRC, 2009) [102]. To achieve a grade of A the research is required to be a high quality randomized control trial (RCT) or a systematic review of high quality RCTs. Laboratory (in vitro) research was classified as level V evidence (DeVries and Berlet, 2010 [103]; The University of Newcastle Australia, 2014 [104]).

A: body of evidence can be trusted to guide practice, systematic review or RCT.

B: body of evidence can be trusted to guide practice in most situations, RCT or high quality observational study.

C: body of evidence provides some support for recommendation but care should be taken in its application, observational studies.

D: Level V evidence or evidence that is weak and recommendation must be applied with caution, expert opinion, animal, or laboratory studies.
